# Supplementary material for: Quantification of antibiotic resistance genes and mobile genetic in dairy manure
Source: PeerJ. 2021 Dec 23;9:e12408. doi: 10.7717/peerj.12408 (PMC8710253; doi:10.7717/peerj.12408)
Supplement: Supplemental Information 1 [file peerj-09-12408-s001.pdf]

SUPPLEMENTAL MATERIAL

Quantification of antibiotic resistance genes and mobile genetic in dairy manure

Yi Wang[1, 2], Pramod Pandey[1], Colleen Chiu[1], Richard Jeannotte[3], Sundaram Kuppu[1], Ruihong Zhang[2], Richard Pereira[1], Bart Weimer[1], Sharif Aly[1]*

[1]Department of Population Health and Reproduction, School of Veterinary Medicine, University of California-Davis, California 95616, USA

[2]Department of Biological and Agricultural Engineering, University of California-Davis, California 95616, USA

[3]Department of Plant Science, University of California Davis

** Corresponding authors:*

Pramod Pandey Email: pkpandey@ucdavis.edu

sulII gene:
CGGGAATGCCATCTGCCTTGAGCGCGTCCAGCACCGGCGCGATACGCGCGATTTCTG
TGTCGGACGAAACAGGCGCGGCGTCGGGATTGCTGGATGCCGGACCGAGGTCGATC
ACATCTGCCCCCTCGGCCATCAGCTTACGCGCCTGCGCAATGGCTGCGTCTGGCGCC
AGATACCGGCCTCCACCGGA

Download   Graphics   Next   Previous   Descriptions

Sequence ID: Query_47145   Length: 190   Number of Matches: 1

Range 1: 1 to 190 Graphics   Next Match   Previous Match

Score	Expect	Identities	Gaps	Strand
346 bits(187)	5e-99	189/190(99%)	0/190(0%)	Plus/Minus

```
Query  1671  TCCGATGGAGGCCGGTATCTGGCGCCAGACGCAGCCATTGCGCAGGCGCGTAAGCTGATG  1730
             |||| |||||||||||||||||||||||||||||||||||||||||||||||||||||||
Sbjct  190   TCCGGTGGAGGCCGGTATCTGGCGCCAGACGCAGCCATTGCGCAGGCGCGTAAGCTGATG  131

Query  1731  GCCGAGGGGGCAGATGTGATCGACCTCGGTCCGGCATCCAGCAATCCCGACGCCGCGCCT  1790
             ||||||||||||||||||||||||||||||||||||||||||||||||||||||||||||
Sbjct  130   GCCGAGGGGGCAGATGTGATCGACCTCGGTCCGGCATCCAGCAATCCCGACGCCGCGCCT  71

Query  1791  GTTTCGTCCGACACAGAAATCGCGCGTATCGCGCCGGTGCTGGACGCGCTCAAGGCAGAT  1850
             ||||||||||||||||||||||||||||||||||||||||||||||||||||||||||||
Sbjct  70    GTTTCGTCCGACACAGAAATCGCGCGTATCGCGCCGGTGCTGGACGCGCTCAAGGCAGAT  11

Query  1851  GGCATTCCCG  1860
             ||||||||||
Sbjct  10    GGCATTCCCG  1
```

Fig. S1. BLAST with *sulII* gene sequence (GenBank: EU360945.1)

tetW gene:
GAGAGCCTGCTATATGCCAGCGGAGCCATTTCAGAACCGGGGAGCGTCGAAAAAGG
GACAACGAGGACGGACACCATGTTTTTGGAGCGGCAGCGTGGGATACCATTCAAGC
GGCAGTCACTTCCTTCCAGTGGCACAGATGTAAAGTTAACATTGTGGATACGCCC

Download   GenBank Graphics   Next   Previous   Descriptions

uncultured bacterium IN-02 tet(W) gene for tetracycline resistance ribosomal protection protein Tet(W), complete CDS

Sequence ID: NG_062353.1   Length: 2120   Number of Matches: 1

Range 1: 161 to 328 GenBank   Graphics   Next Match   Previous Match

Score	Expect	Identities	Gaps	Strand
303 bits(164)	1e-78	167/168(99%)	1/168(0%)	Plus/Plus

```
Query  1    GAGAGCCTGCTATATGCCAGCGGAGCCATTTCAGAACCGGGGAGCGTCGAAAAAGGGACA  60
            ||||||||||||||||||||||||||||||||||||||||||||||||||||||||||||
Sbjct  161  GAGAGCCTGCTATATGCCAGCGGAGCCATTTCAGAACCGGGGAGCGTCGAAAAAGGGACA  220

Query  61   ACGAGGACGGACACCATGTTTTTGGAGCGGCAGCGTGGGA-TACCATTCAAGCGGCAGTC  119
            |||||||||||||||||||||||||||||||||||||||| |||||||||||||||||||
Sbjct  221  ACGAGGACGGACACCATGTTTTTGGAGCGGCAGCGTGGGATTACCATTCAAGCGGCAGTC  280

Query  120  ACTTCCTTCCAGTGGCACAGATGTAAAGTTAACATTGTGGATACGCCC  167
            ||||||||||||||||||||||||||||||||||||||||||||||||
Sbjct  281  ACTTCCTTCCAGTGGCACAGATGTAAAGTTAACATTGTGGATACGCCC  328
```

Fig. S2. BLAST with *tetW* gene sequence (NCBI Reference Sequence: NG_062353.1)

intI1 gene:
ACATGCGTGTAAATCATCGTCGTGGAAACGTCGGAATGGCCGAGTAGGTCCTGCAC
GGTTCGAATGTCGTAACCGCTGCGGAGCAAGGCCGTCGCGAACGAGTGGCGGAGGG
TGTGCGGTGTGGCGGGCTTCGTGATGCCTGCTTGTTCTACGGCACGTTTGAAGGCGC
GCTGAAAGGTCTGGTCATACATGTGATGGCGACGCACGACACCGCTCCGTGGATCG
GTCGAATGCGTGTGCTGCGCGAAAACCCAGAACCACGGCCAAGAATGCCCGGCGCG
CGGATACTTCCGCTCAAGGGCGTCGGGAAGCGCAACGCCGCTGCGGCCCTCGGCCT
GGTCCTTCAGCCACCATGCCCGTGCACGCGACAGCTGCTCGCGCAGGCTGGGTGCCA
AGCTCTCGGGTAACATCAAGGCCCGATCCTTGGAGCCCTTGCCTTCCCGCACGATGA
TCGTGCCGTGATCGAAATCCAG

Download GenBank Graphics ▼ Next ▲ Previous ◀ Descriptions

Pseudomonas sp. 9SN1 Class 1 integron integrase (intI1) gene, partial cds

Sequence ID: JN870910.1 **Length:** 944 **Number of Matches:** 1

Range 1: 1 to 473 GenBank Graphics ▼ Next Match ▲ Previous Match

Score	Expect	Identities	Gaps	Strand
841 bits(455)	0.0	467/473(99%)	0/473(0%)	Plus/Plus

```
Query  1    ACATGCGTGTAAATCATCGTCGTGGAAACGTCGGAATGGCCGAGTAGGTCCTGCACGGTT  60
            ||||||||||||||||||||||| || ||||||||||||||||| || ||||||||||||
Sbjct  1    ACATGCGTGTAAATCATCGTCGTAGAGACGTCGGAATGGCCGAGCAGATCCTGCACGGTT  60

Query  61   CGAATGTCGTAACCGCTGCGGAGCAAGGCCGTCGCGAACGAGTGGCGGAGGGTGTGCGGT  120
            ||||||||||||||||||||||||||||||||||||||||||||||||||||||||||||
Sbjct  61   CGAATGTCGTAACCGCTGCGGAGCAAGGCCGTCGCGAACGAGTGGCGGAGGGTGTGCGGT  120

Query  121  GTGGCGGGCTTCGTGATGCCTGCTTGTTCTACGGCACGTTTGAAGGCGCGCTGAAAGGTC  180
            ||||||||||||||||||||||||||||||||||||||||||||||||||||||||||||
Sbjct  121  GTGGCGGGCTTCGTGATGCCTGCTTGTTCTACGGCACGTTTGAAGGCGCGCTGAAAGGTC  180

Query  181  TGGTCATACATGTGATGGCGACGCACGACACCGCTCCGTGGATCGGTCGAATGCGTGTGC  240
            ||||||||||||||||||||||||||||||||||||||||||||||||||||||||||||
Sbjct  181  TGGTCATACATGTGATGGCGACGCACGACACCGCTCCGTGGATCGGTCGAATGCGTGTGC  240

Query  241  TGCGCGAAAACCCAGAACCACGGCCAAGAATGCCCGGCGCGCGGATACTTCCGCTCAAGG  300
            ||||| ||||||||||||||||||||||||||||||||||||||||||||||||||||||
Sbjct  241  TGCGCAAAAACCCAGAACCACGGCCAAGAATGCCCGGCGCGCGGATACTTCCGCTCAAGG  300

Query  301  GCGTCGGGAAGCGCAACGCCGCTGCGGCCCTCGGCCTGGTCCTTCAGCCACCATGCCCGT  360
            ||||||||||||||||||||||||||||||||||||||||||||||||||||||||||||
Sbjct  301  GCGTCGGGAAGCGCAACGCCGCTGCGGCCCTCGGCCTGGTCCTTCAGCCACCATGCCCGT  360

Query  361  GCACGCGACAGCTGCTCGCGCAGGCTGGGTGCCAAGCTCTCGGGTAACATCAAGGCCCGA  420
            ||||||||||||||||||||||||||||||||||||||||||||||||||||||||||||
Sbjct  361  GCACGCGACAGCTGCTCGCGCAGGCTGGGTGCCAAGCTCTCGGGTAACATCAAGGCCCGA  420

Query  421  TCCTTGGAGCCCTTGCCTTCCCGCACGATGATCGTGCCGTGATCGAAATCCAG  473
            ||||||||||||||||| |||||||||||||||||||||||||||||||||||
Sbjct  421  TCCTTGGAGCCCTTGCCCTCCCGCACGATGATCGTGCCGTGATCGAAATCCAG  473
```

Fig. S3. BLAST with *intI1* gene sequence (GenBank: JN870910.1)

ermF gene
CGACACAGCTTTGGTTGAACATTTACGAAAATTATTTTCTGATGCCCGAAATGTTCA
AGTTGTCGGTTGTGATTTTAGGAATTTAGCAGTTCCGAAATTTCCTTTCAAAGTGGTG
TCAAATATTCCTTATGGCATTACTTCCGATATTTTCAAAATCCTGATGTTTGAGAGTC
TTGGAAATTTTCGGGGAGGTTCCATTGTCCTTCAGTTAGAACCTACACAAAAGTTAT
TTTCGAGGAAGCTTTACAATCCATATACCGTTTTCTATCATACTTTTTTTGATTTGAA
ACTTGTCTATGAGGTAGGTCC

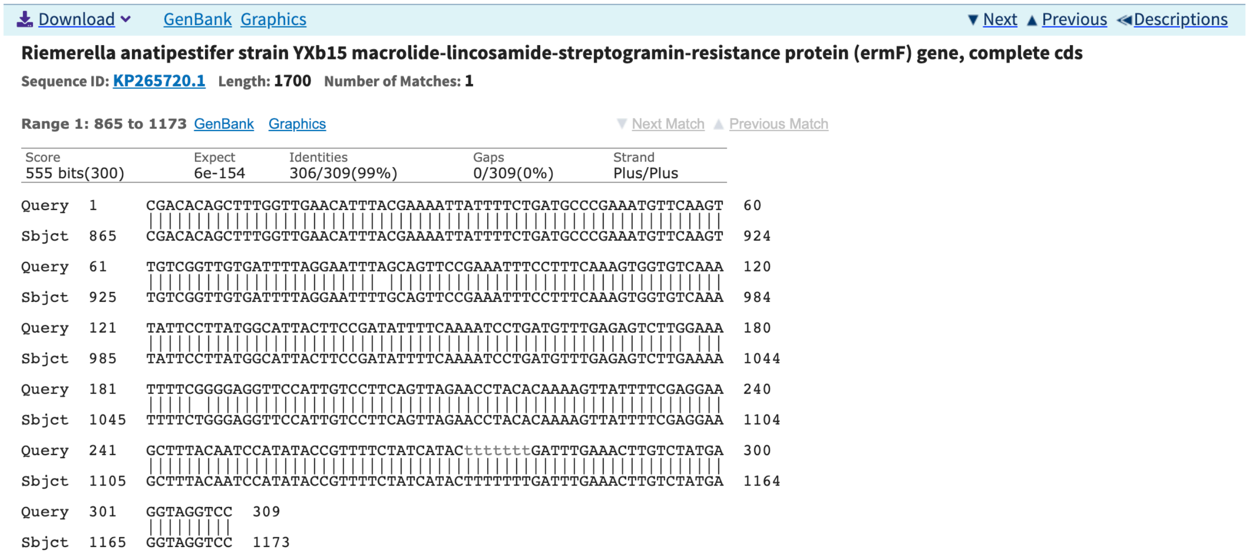

Download GenBank Graphics ▼ Next ▲ Previous ◀ Descriptions

Riemerella anatipestifer strain YXb15 macrolide-lincosamide-streptogramin-resistance protein (ermF) gene, complete cds

Sequence ID: KP265720.1 Length: 1700 Number of Matches: 1

Range 1: 865 to 1173 GenBank Graphics ▼ Next Match ▲ Previous Match

Score	Expect	Identities	Gaps	Strand
555 bits(300)	6e-154	306/309(99%)	0/309(0%)	Plus/Plus

```
Query  1     CGACACAGCTTTGGTTGAACATTTACGAAAATTATTTTCTGATGCCCGAAATGTTCAAGT  60
             ||||||||||||||||||||||||||||||||||||||||||||||||||||||||||||
Sbjct  865   CGACACAGCTTTGGTTGAACATTTACGAAAATTATTTTCTGATGCCCGAAATGTTCAAGT  924

Query  61    TGTCGGTTGTGATTTTAGGAATTTAGCAGTTCCGAAATTTCCTTTCAAAGTGGTGTCAAA  120
             |||||||||||||||||||||||| |||||||||||||||||||||||||||||||||||
Sbjct  925   TGTCGGTTGTGATTTTAGGAATTTTGCAGTTCCGAAATTTCCTTTCAAAGTGGTGTCAAA  984

Query  121   TATTCCTTATGGCATTACTTCCGATATTTTCAAAATCCTGATGTTTGAGAGTCTTGGAAA  180
             |||||||||||||||||||||||||||||||||||||||||||||||||||||||| |||
Sbjct  985   TATTCCTTATGGCATTACTTCCGATATTTTCAAAATCCTGATGTTTGAGAGTCTTGAAAA  1044

Query  181   TTTTCGGGGAGGTTCCATTGTCCTTCAGTTAGAACCTACACAAAAGTTATTTTCGAGGAA  240
             ||||| ||||||||||||||||||||||||||||||||||||||||||||||||||||||
Sbjct  1045  TTTTCTGGGAGGTTCCATTGTCCTTCAGTTAGAACCTACACAAAAGTTATTTTCGAGGAA  1104

Query  241   GCTTTACAATCCATATACCGTTTTCTATCATACtttttttGATTTGAAACTTGTCTATGA  300
             ||||||||||||||||||||||||||||||||||||||||||||||||||||||||||||
Sbjct  1105  GCTTTACAATCCATATACCGTTTTCTATCATACTTTTTTTGATTTGAAACTTGTCTATGA  1164

Query  301   GGTAGGTCC  309
             |||||||||
Sbjct  1165  GGTAGGTCC  1173
```

Fig.S4. BLAST with *ermF* gene sequence (GenBank: KP265720.1)

tnpA gene
CCGATCACGGAAAGCTCAAGATACTGATCAGGCCGGTGCGCAGTTTCAAATCGATC
CCCACGGCCTATGCCACGATCAAGGGATTCGAAGTCATGCGAGCC

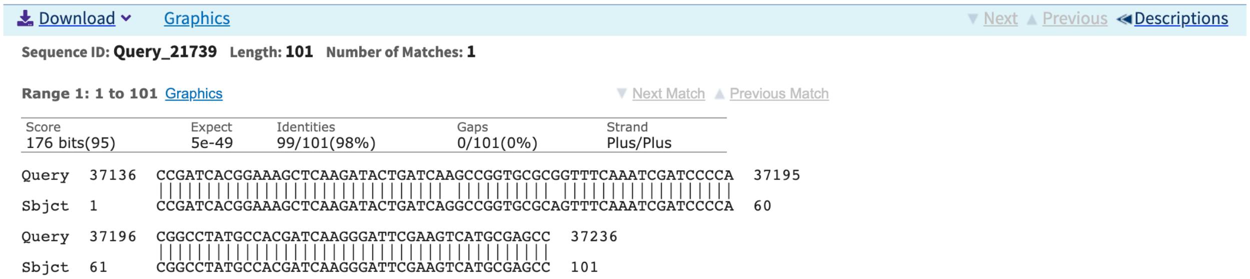

Fig. S5. BLAST with *tnpA* gene sequence (Gene ID: 15152808)

16S rRNA gene:
ATTACCGCGGCTGCTGGCACGAAGTTAGCCGGTGCTTATTCTTTGGGTACCGTCAGA
ACAACCGGGTATTAGCCGGCTGCTTTTCTTTCCCAACAAAAGGGCTTTACAACCCGA
AGGCCTTCTTCACCCACGCGGTATGGCTGGATCAGGCTTGCGCCCATTGTCCAATAT
TCCCCACTGCTGCCTCCCGTAGG

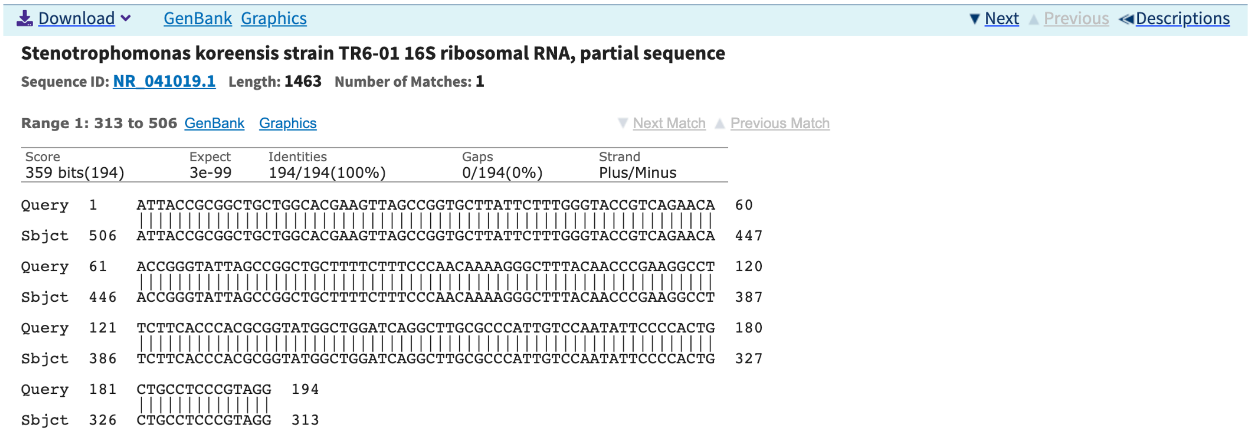

Fig. S6. BLAST with 16S rRNA gene sequence (NCBI Reference Sequence: NR_041019.1)
