# Supplementary material for: Quantification of antibiotic resistance genes and mobile genetic in dairy manure
Source: PeerJ. 2021 Dec 23;9:e12408. doi: 10.7717/peerj.12408 (PMC8710253; doi:10.7717/peerj.12408)
Supplement: Supplemental Information 2 [file peerj-09-12408-s002.docx]

**Table S1 (supplementary) qPCR results for 33 dairy samples.** Gene concentrations were normalized to the number of copies of bacterial 16S rRNA gene. Underlined data were beyond lower outer fence: Q1 – 3 IQ and upper outer fence: Q3 + 3 IQ. They were considered as extreme outliers and removed from the table for average calculation and statistical analysis.

|  | Sample ID | treatment | *sulII* | *tetW* | *ermF* | *intI1* | *tnpA* |  |  |
| --- | --- | --- | --- | --- | --- | --- | --- | --- | --- |
| 1 | FP1 | FP | 8.40E-04 | 2.10E-05 | 7.58E-06 | 4.81E-04 | 2.23E-05 |  |  |
| 2 | FP2 | FP | 4.16E-05 | 1.07E-06 | 3.15E-08 | 2.52E-05 | 8.12E-06 |  |  |
| 3 | FP3 | FP | 9.32E-07 | 3.79E-08 | 2.18E-05 | 2.62E-06 | 4.79E-07 |  |  |
| 4 | FP4 | FP | 4.71E-05 | 2.84E-05 | 1.45E-09 | 1.98E-04 | 1.71E-04 |  |  |
| 5 | FP5 | FP | 2.01E-03 | 2.11E-04 | 1.26E-05 | 1.09E-04 | 2.08E-04 |  |  |
| 6 | FP6 | FP | 1.31E-04 | 3.82E-04 | 9.67E-06 | 1.04E-04 | 1.89E-05 |  |  |
| 7 | FP7 | FP | 3.08E-04 | 1.15E-04 | 6.11E-06 | 2.13E-04 | 5.16E-05 |  |  |
| 8 | CP1 | CP | 4.44E-06 | 6.49E-08 | 2.03E-07 | 8.53E-06 | 7.90E-06 |  |  |
| 9 | CP2 | CP | 1.61E-06 | 1.14E-07 | 1.13E-05 | 1.50E-06 | 1.41E-07 |  |  |
| 10 | CP3 | CP | 4.18E+00 | 6.27E-02 | 0.00E+00 | 6.14E-02 | 1.88E+01 |  |  |
| 11 | CP4 | CP | 2.93E-04 | 1.61E-05 | 1.96E-06 | 2.43E-04 | 1.37E-05 |  |  |
| 12 | CP5 | CP | 6.59E-05 | 2.32E-05 | 3.45E-09 | 5.37E-05 | 4.79E-06 |  |  |
| 13 | CP6 | CP | 9.21E-05 | 2.85E-05 | 5.84E-07 | 8.33E-05 | 4.40E-05 |  |  |
| 14 | CP7 | CP | 6.00E-04 | 7.01E-06 | 2.16E-08 | 3.62E-05 | 4.34E-04 |  |  |
| 15 | FM1 | FM | 1.01E-04 | 6.92E-04 | 5.36E-06 | 2.65E-05 | 2.58E-05 |  |  |
| 16 | FM2 | FM | 3.11E-05 | 1.42E-04 | 1.40E-06 | 9.02E-06 | 1.59E-05 |  |  |
| 17 | FM3 | FM | 3.08E-05 | 1.33E-04 | 3.62E-06 | 2.32E-05 | 1.65E-05 |  |  |
| 18 | FM4 | FM | 2.22E-04 | 2.21E-04 | 1.23E-06 | 8.08E-06 | 1.07E-04 |  |  |
| 19 | FM5 | FM | 1.27E-04 | 1.91E-03 | 4.21E-06 | 1.40E-05 | 1.62E-04 |  |  |
| 20 | FM6 | FM | 1.53E-04 | 5.20E-04 | 1.31E-06 | 2.58E-05 | 9.91E-05 |  |  |
| 21 | PL1 | PL | 1.75E-04 | 1.13E-04 | 2.03E-05 | 3.90E-04 | 4.25E-06 |  |  |
| 22 | PL2 | PL | 4.56E-04 | 2.43E-03 | 0.00E+00 | 2.54E-04 | 1.75E-04 |  |  |
| 23 | PL3 | PL | 6.71E-05 | 5.30E-05 | 1.17E-05 | 1.58E-05 | 1.76E-05 |  |  |
| 24 | PL4 | PL | 5.31E-05 | 6.81E-05 | 9.51E-06 | 1.43E-05 | 3.12E-05 |  |  |
| 25 | PL5 | PL | 4.25E-05 | 1.06E-04 | 6.58E-06 | 1.15E-05 | 1.63E-05 |  |  |
| 26 | PL6 | PL | 6.93E-05 | 2.04E-04 | 1.33E-06 | 4.92E-06 | 2.52E-05 |  |  |
| 27 | PL7 | PL | 1.42E-04 | 3.25E-04 | 3.78E-06 | 2.39E-05 | 6.81E-05 |  |  |
| 28 | PL8 | PL | 5.17E-05 | 5.73E-04 | 1.54E-06 | 5.80E-06 | 4.11E-05 |  |  |
| 39 | SL1 | SL | 1.75E-04 | 2.69E-05 | 7.95E-07 | 4.70E-04 | 3.14E-05 |  |  |
| 30 | SL2 | SL | 4.11E-05 | 1.08E-04 | 1.45E-05 | 1.51E-05 | 4.71E-06 |  |  |
| 31 | SL3 | SL | 2.94E-05 | 3.04E-05 | 3.50E-05 | 1.78E-05 | 2.23E-06 |  |  |
| 32 | SL4 | SL | 3.78E-05 | 8.71E-06 | 2.13E-06 | 1.51E-05 | 4.36E-06 |  |  |
| 33 | SL5 | SL | 3.33E-05 | 6.62E-03 | 1.34E-06 | 6.13E-04 | 1.44E-03 |  |  |
| Average | | | 1.04E-04 | 1.43E-04 | 5.98E-06 | 1.10E-04 | 4.67E-05 |  |  |
